# Volumetric Change Detection in Bedrock Coastal Cliffs Using Terrestrial Laser Scanning and UAS-Based SfM

**DOI:** 10.3390/s20123403

**Published:** 2020-06-16

**Authors:** Yuichi S. Hayakawa, Hiroyuki Obanawa

**Affiliations:** 1 Faculty of Environmental Earth Science, Hokkaido University, Sapporo 060-0810, Japan; 2 Hokkaido Agricultural Research Center, National Agriculture and Food Research Organization, Sapporo 062-8555, Japan; obanawah924@affrc.go.jp

**Keywords:** coastal erosion, terrestrial laser scanning, structure from motion, photogrammetry, unmanned aerial system, point cloud, change detection

## Abstract

Three-dimensional (3D) morphological changes in rocky coasts need to be precisely measured for protecting coastal areas and evaluating the associated sediment dynamics, although volumetric measurements of bedrock erosion in rocky coasts have been limited due to the lack of appropriate measurement methods. Here we carried out repeat surveys of the 3D measurements of a small coastal island using terrestrial laser scanning (TLS) and structure-from-motion (SfM) photogrammetry with an unmanned aerial system (UAS) for 5 years. The UAS-SfM approach measures the entire shape of the island, whereas the TLS measurement enables to obtain more accurate morphological data at a scale of centimeters on the land side. The multitemporal TLS-derived data were first aligned in timeline by the iterative closest point (ICP) method and they were used as positionally correct references. The UAS-SfM data were then aligned to each of the TLS-derived data by ICP to improve its positional accuracy. The changed areas for each period was then extracted from the aligned UAS-derived point clouds and were converted to 3D mesh polygons, enabling a differential volume estimate (DVE). The DVE for each period was revealed to be from 3.1 to 77.2 m^3^/month. These changes are rapid enough to force the coastal bedrock island to disappear in 30 years. The temporal variations in the DVE is roughly associated with those in the frequency of high tidal waves.

## 1. Introduction

In response to regional sea-level rise or local land subsidence, coastal erosion is a significant issue to be mitigated both in sandy and rocky coasts [1,2,3]. In particular, erosion along rocky coasts may have larger impact on the degradation of land areas because of its irreversible processes [4,5]. For this reason, a considerable fraction of vulnerable rocky coasts with erodible lithology is artificially protected by modern works with a huge investment, and the erosion rate of such rocky coasts has notably decreased over time, although its long-term effects need to be carefully assessed [6,7]. Moreover, the decrease in cliff erosion limits the sand supply to the nearby sandy beach, where coastal erosion is now another considerable problem, including sand exhaustion in nearby beaches [8]. The connected dynamics of cliff erosion and sand deposition in rocky and sandy coasts needs to be mutually examined. A quantitative evaluation of bedrock erosion rates by natural processes would provide a significant insight into the sediment dynamics in the coastal system [9].

However, conventional approaches of erosion measurement in rocky coasts are limited in terms of their accessibility and accuracy with three-dimensional (3D) measurements, while most of the studies have adopted two-dimensional approaches using historical aerial photographs or airborne laser-scanning data [10,11,12]. In particular, a detailed shape of the sea side of overhanging coastal cliffs has often been difficult to measure in 3D because of the lack of platforms of measurement. Recent technological development in the use of unmanned aerial systems (UASs), structure-from-motion (SfM) multi-view stereo photogrammetry, and terrestrial laser scanning (TLS) enabled us to overcome the difficulties in measuring inaccessible areas with a certain accuracy [13,14,15,16]. UAS is often combined with SfM photogrammetry so that the close-range aerial photographs can be utilized to generate a 3D point cloud of geomorphological objects [14,15]. TLS can provide a dense and accurate 3D point cloud of target objects with spatially random laser returns [13,16,17,18]. These technologies are useful but not fully applied to the evaluation of rocky coast erosion, probably because of insufficient abilities of each of the methods. TLS is often accurate enough to detect centimeter-scale changes in coastal cliffs [13,16,17,18,19,20], but the area of measurement is limited due to the limited areas of its placement along the coast. UAS-based photogrammetry is often capable of measuring wider, inaccessible areas unlike the ground-based surveys, although its accuracy is generally lower than that by laser scanning if without ground control points (GCPs) [15,21].

Here we propose a combined use of TLS and UAS-based photogrammetry to monitor 3D changes in erodible coastal cliffs. For measurements of landforms having complex 3D morphology, the combination of multiple sensors is advantageous in addressing the disadvantages of the different methods: limited measurement areas by TLS and lower accuracies by UAS-photogrammetry. With the limited availability of setting GCPs in inaccessible coastal cliffs, more accurate TLS-derived data that are capable of representing a part of cliffs are used as the reference instead of setting ground control points in UAS-derived data. The use of such different sensors of both TLS and UAS is often challenged in different fields of studies including 3D modeling, cultural heritage, and forestry [22,23,24], but in those cases, TLS and UAS were used as complementary to each other to cover the invisible areas from ground or air. The approach of this study is different from the previous ones in that the TLS-derived data are only used as the reference for UAS-derived data instead of GCPs.

Furthermore, the detailed assessments of the obtained 3D data enable us to reveal detailed temporal changes in the 3D shape of bedrock coasts. The proposed method is new in terms of using 3D polygons for volumetric analysis, while point cloud comparisons without volumetric measurements have often been proposed [25].

## 2. Materials and Methods

### 2.1. Study Site

**Figure 1:**
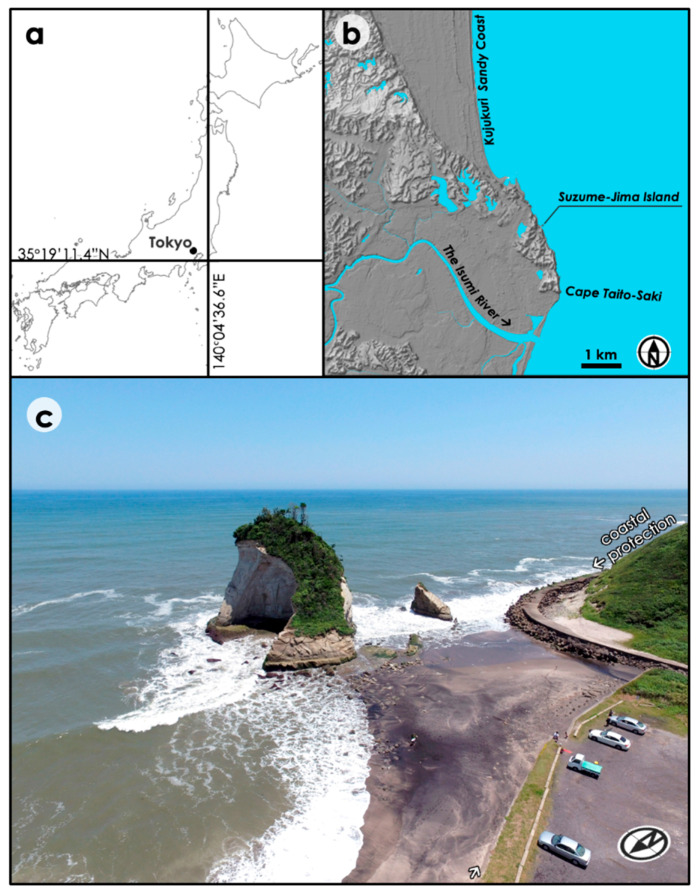
Study site: the Suzume-Jima island. (**a**) Overview map; (**b**) hillshade image around the study site. The Kujukuri Sandy Coast continues to the north for 66 km, whereas the rocky coasts appear in the southern side; (**c**) the aerial view of the Suzume-Jima Island (taken on 18 June 2016). Note that the island is located outside of the protection along the coast.

Bedrock cliffs of a small coastal island in the outer Boso Peninsula (eastern Japan) is an ideal experimental site for this study (Figure 1). The island, named Suzume-Jima Island, has a conical, circular shape with a diameter of 50 m and a height of 30 m. The western side of the island is connected to the land during low tides, but the slopes around the island are too steep to be physically accessed. The island’s sea side is therefore not visible and cannot be measured from the land side. The total area of the vertically projected areas for all the bedrock faces (west, south, east, and north sides) of the island is 1,486 m^2^. The area is characterized by early to middle Pleistocene (Gelasian) sedimentary rocks, including weak sandstones and mudstones of Kiwada Formation [26]. The mean annual erosion rates of coastal cliffs in the area have been reported to be over 1 m for centuries based on 1:1000 topographic maps during the period of 1960–1966 [1,10]. Most portions of the cliffs have been protected by artificial embankment along the rocky coast for decades, but this island is located outside the protection so that natural processes of erosion are still observable. Offshore wave heights are estimated by the Japan Meteorological Agency (JMA) every 12 h near the site (Offshore Boso Peninsula: 35°20′, 140°45′). The average wave height in the last 5 years was 1.94 m with a standard deviation of 0.85 m. The climate of the area is characterized by 1969.7 mm of mean annual precipitation, 6.6–25.6 °C of mean monthly temperature, 2.7–4.1 m/y of mean monthly wind speed, and 1920.5 h of mean annual sunshine duration (JMA Automated Meteorological Data Acquisition System observation site at Katsuura: 35°9.0′, 140° 18.7′, 12 m a.s.l., the average for 1981–2010).

Obanawa and Hayakawa [27] preliminarily analyzed this site with data by repeated measurements for two years. The average erosion volume was revealed to be 24.2 m^3^/month from June 2014 to June 2016, whose data were also integrated in this study. It was concluded that impacts by wavecuts dominates in the bedrock erosion and rockfalls rather than remarkable earthquakes.

### 2.2. Measurements Using TLS and UAS-SfM

**Figure 2:**
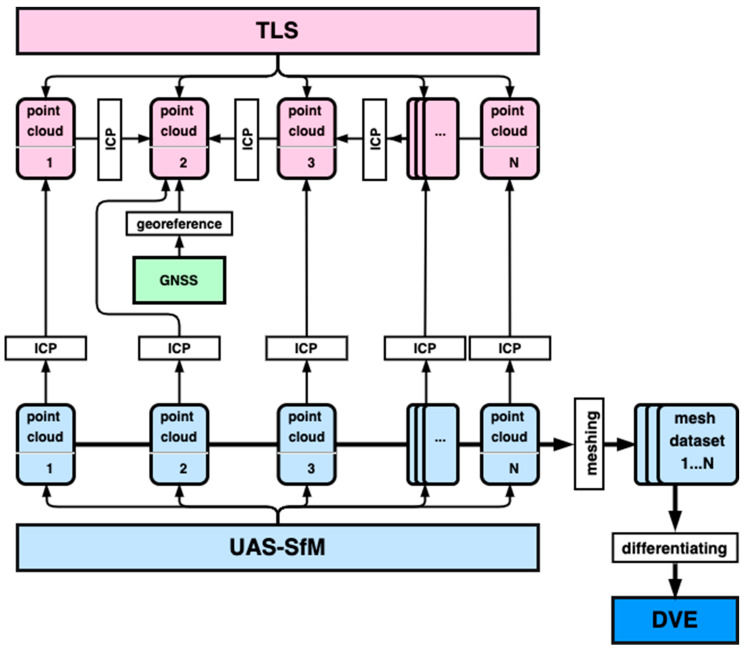
Workflow of the methodology in this study. TLS: Terrestrial Laser Scanning, UAS: Unmanned Aerial System, SfM: Structure-from-Motion, GNSS: Global Navigation Satellite System, ICP: Iterative Closest Point, DVE: Differential Volume Estimate..

We carried out measurements of the 3D morphology of the Suzume-Jima Island with the combined use of TLS and UAS-based SfM photogrammetry every 3 to 6 months for 5 years. The land-side of the island was measured with TLS (Topcon GLS-1500 and Trimble TX5), whereas the entire shape of the island including the inaccessible seaside was measured using UAS-SfM. For the UAS measurements, DJI Phantom 2, Phantom 3 Pro, Phantom 4, or Mavic 2 Pro was used. The in-built digital cameras were used for Phantom 3, Phantom 4, and Mavic 2, while an external digital camera (Nikon COOLPIX A) was mounted on Phantom 2. The obtained point cloud data by TLS were initially processed with the bundle software (Trimble RealWorks 8.1) to perform internal registrations of the point clouds from different scan positions, generating one-point cloud dataset for each measurement time. The internal registration errors were measured as the root mean square (RMS) of the mean spacings between the closest points in the aligned point clouds [28]. Low-altitude (30–80 m a.s.l.) oblique aerial images taken by the UAS were processed by a SfM photogrammetry software Agisoft Metashape (formerly PhotoScan) to generate a dense point cloud of the entire island. Although the geographical positions of the point cloud are roughly given from the image-based locations taken by an in-built global navigation satellite system (GNSS) of the aircraft, the accuracy of the positions is often worse than 1 m due to the errors of the single-point positioning of the aircraft GNSS. Figure 2 shows the entire workflow of the methodology.

The data by TLS were used as a reference to match the temporal point cloud datasets with a better spatial accuracy (at millimeter to centimeter levels) than those by the UAS-SfM (at centimeter to decimeter levels) [9,13,20,21]. The land-side TLS point cloud data for each measurement time were aligned to the nearest time dataset, one of which was fixed as the basic reference whose coordinates were obtained using ground control points (GCPs). The accurate (at millimeter to centimeter levels) geographic coordinates of the GCPs were obtained by post-processing static measurements by the GNSS. A rover with an antenna (Trimble Geo7 receiver with Zephyr2 antenna) was used to measure each position of the GCPs and the data from the reference station (Chiba-Ohara, 140°23′05.6739″ E, 35°14′35.6759″ N, operated by the Geospatial Authority of Japan) were used to perform post-processed kinematic (PPK) correction. Five GCPs were picked up during the TLS-derived point cloud measurement on February 23, 2016, and the coordinates derived from the PPK-GNSS were assigned to the GCPs in the point cloud data to make the point cloud georeferenced. The other TLS point cloud data were then successively aligned to the pre- or post-change point cloud at the nearest time using the iterative closest point (ICP) method [29,30]. This method is advantageous in matching temporal datasets of point clouds with accuracies better than those by control points whose coordinates are obtained by GNSS. Unchanged objects such as coastal embankments and the stable slope faces of the island were manually extracted in two point clouds, and one point cloud was roughly aligned to the control point cloud by visual inspection. After automatically extracting overlapping areas, the ICP algorithm was applied to carry out the external registration by minimizing the distances between the nearest points, for which Trimble RealWorks was used. The external registration errors were measured as the root mean square of the mean spacings between the closest points in the aligned point clouds.

**Figure 3:**
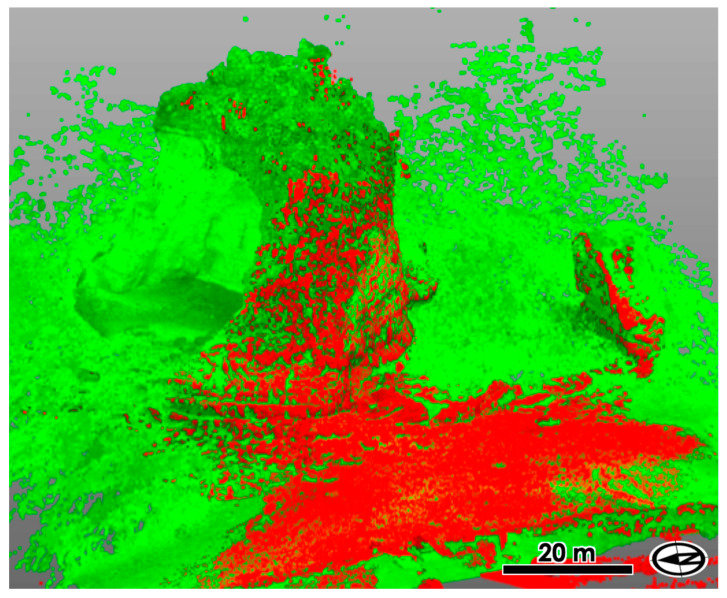
Aligned point clouds by TLS and UAS. Red points indicate those taken by TLS, whereas green points are those by UAS-SfM. The TLS-derived point cloud is only available for the land side. The UAS-derived point cloud contains erroneous points by moving sea water, which were removed after the alignment.

The UAS-derived point clouds were then aligned to the registered TLS point clouds for each measurement time (Figure 3). The ICP method was again applied for these alignments using Trimble RealWorks. The positional accuracies of the entire point cloud by UAS was improved based on the positions of TLS point clouds on the land side of the island. The registration errors of UAS-derived point clouds to TLS-derived point clouds were measured as the RMS of the mean spacings between the closest points in the aligned point clouds. We adopted this approach because the possible locations of GCPs for the UAS-derived data were quite limited in the land-side area, away from the island. We needed extensive locations on the island to place if the GCPs were to be placed [31], but the accessibility was quite limited.

For the following analyses, a free and open source software CloudCompare was used for the manipulation of the point cloud data [32]. First, surface normal directions were assigned to each point in the point clouds by setting a local model surface to the neighboring points. This process was necessary to identify the front and back sides of the surface represented by the point cloud, and for generating meshes with connected faces of points. The normal direction was calculated based on the barycenter of the island as the origin of preferred orientation, so that the bedrock surface was correctly identified as the front side.

**Figure 4:**
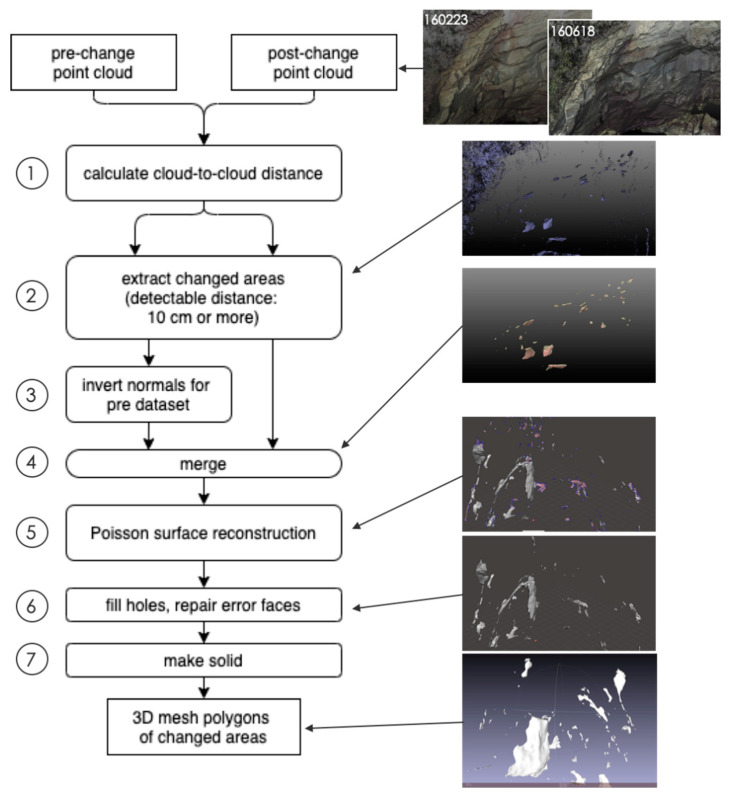
Flow chart of the differential volume estimate (DVE) processing. The differentiation process is given in the left side, while the right-side pictures show the screenshots of the data at some key processes.

Differentiation between the point clouds at different measurement times was then performed with the aligned UAS point clouds, which we called differential volume estimate (DVE) (Figure 4). To detect significant changes, the cloud-to-cloud distance was first calculated for both the pre- and post-change point clouds using the CloudCompare software (step 1 in Figure 4), and the points having a certain distance (in this case, more than twice of the maximum registration errors) were extracted from both datasets (step 2). The normal direction of the extracted point cloud of the pre-change dataset was then inversed (step 3). The inversion of the surface orientation for the pre-change dataset enabled the identification of the surface of missing mass, not the surface of remaining bedrock cliffs. Merging the extracted points of the pre-change (inverse surface) and post-change (original surface) point clouds, the missing area can be expressed as point clouds with appropriate normal directions (step 4). Here, point clouds in vegetated areas were removed. The remaining point clouds showing feasible changes of bedrock area were then converted to 3D mesh polygons by applying the Poisson surface reconstruction method [33] (step 5). Since the generated 3D mesh polygons contained various errors including holes, non-manifold edges, and self-intersecting surfaces, those errors were fixed using the error check and repair function in the 3D modeling software (Autodesk Meshmixer) (steps 6). The cleaned 3D mesh polygons showing only the surface of rocks were then converted to solid polygons (step 7). The total volume of the eroded areas was calculated from the solid mesh polygon data for each period.

## 3. Results

### 3.1. Field Measurement

Field campaigns were performed 15 times for every 3–6 months from June 2014 to October 2019. Table 1 shows a summary of the field surveys’ results. Periods between the two adjacent measurements were named as I–XIV (Table 1). TLS and UAS datasets were taken for every field survey, except for the cases of No. 2, 12, and 14 when the TLS was not available due to machine troubles. For these cases, the UAS-derived point clouds were directly aligned to the UAS point cloud of the pre-change dataset. The number of scan positions of TLS measurements was from 1 to 10, for which the internal registration errors varied from 2.7 to 31.5 mm. The GNSS measurement was performed at the sixth survey (23 February 2016). PPK positioning was carried out for six GCPs, and the georeferencing error for the TLS point cloud based on the ground control points was 4.2 mm.

### 3.2. Point Cloud Alignments

Based on the georeferenced TLS data for the sixth survey (February 23, 2016), ICP registrations of each TLS point cloud were applied in a timeline at errors of 6.4–25.0 mm. Each UAS point cloud was then aligned to each TLS point cloud of the same date, showing mean distances of 18.8–39.7 mm. As mentioned, for the three datasets missing TLS data (October 31, 2014, January 27, 2018, and March 11, 2019), the UAS point clouds were aligned directly to the pre-change or post-change UAS point cloud with errors of 18.8–35.9 mm (Table 1).

### 3.3. Differential Volume Estimate

**Figure 5:**
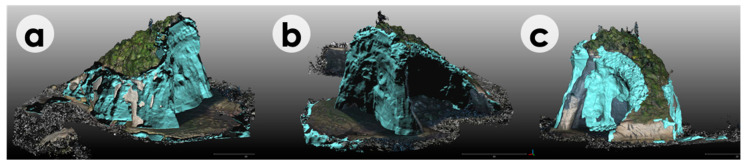
DVE for the entire period showing the total change of the island from 24 June 2014, to 2 October 2019. The total volume loss during this period was 1979.5 m^3^. (**a**) The southeast side; (**b**) the northeast side; and (**c**) the west side.

**Figure 6:**
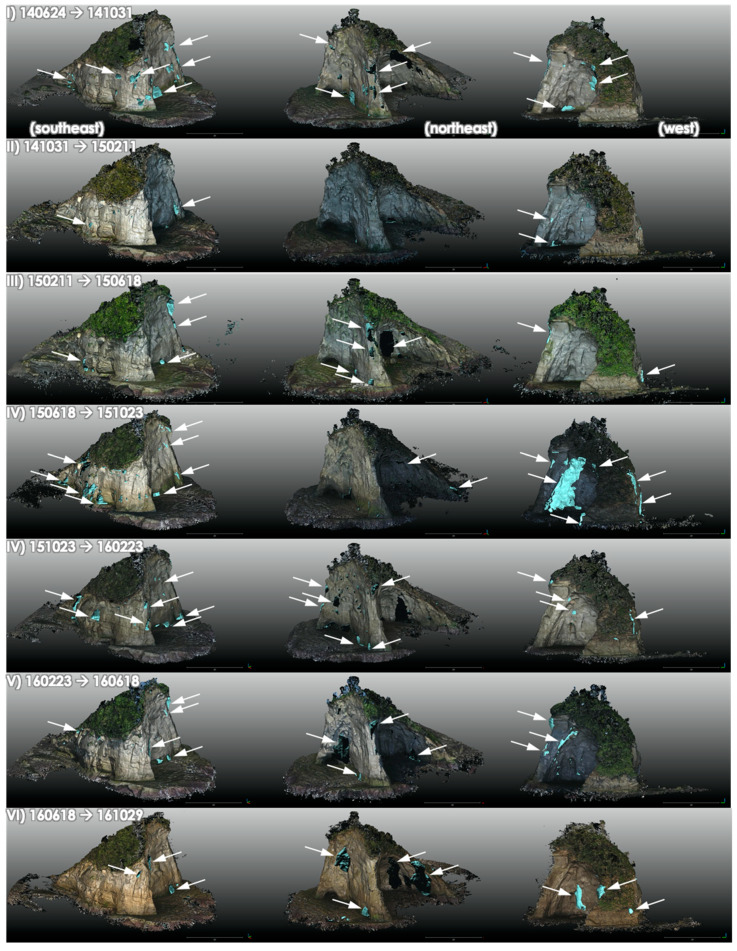

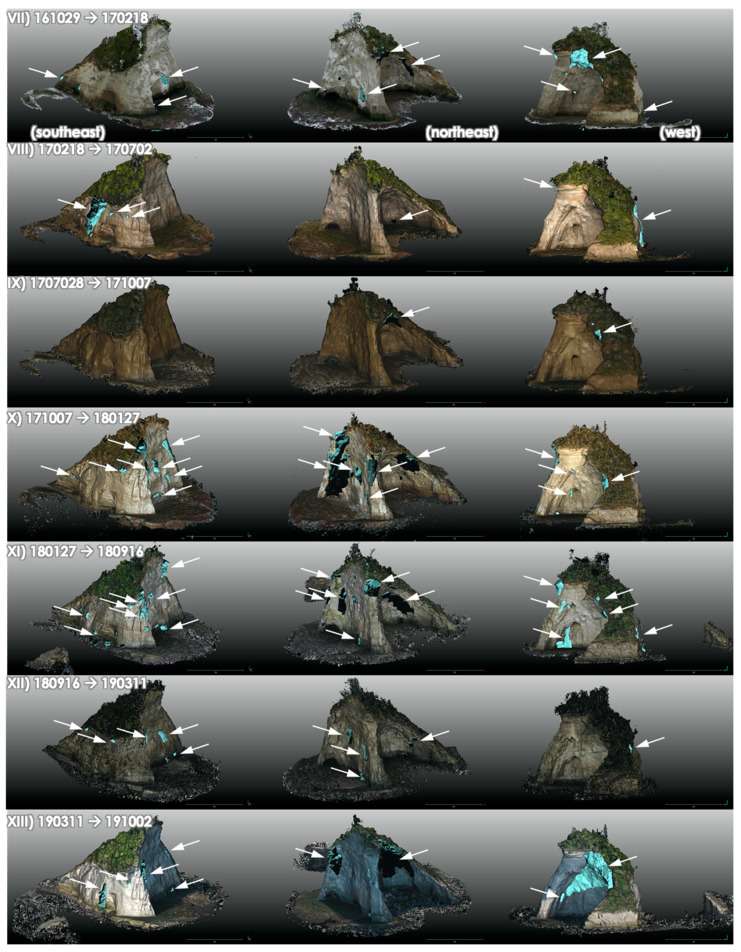
Differential volume estimate (DVE) for each time period, showing the eroded area of the bedrock cliffs by comparing the point clouds taken in different time periods. Changed areas are highlighted with white arrows.

Using the aligned pre- and post-change point clouds, 3D mesh polygons were generated from the points having differences, and the DVE was applied for each period. Among the registration errors of the internal alignments of TLS point clouds, external alignments of pre- and post-change point clouds by TLS, and UAS to TLS point cloud alignments, the maximum registration error was 39.7 mm (Table 1). The minimum distance of the two-point clouds to be detected as significant changes were then defined to be 8 cm (twice that of the maximum registration errors). The total volume loss of the island bedrock was 1979.5 m^3^ (Table 1). The changed areas, either by rockfalls or wave erosion, were revealed to be spatially variable (Figure 5 and Figure 6). The greatest change was observed in the eastern sea-side of the island (Figure 5a), whereas the western land-side slope showed the least changes (Figure 5b). Rockfalls in the cave-like area in the northern side were also frequent, likely due to the gravitational instability of the ceiling of the cave-like cut (Figure 5c).

The eroded volume for each period temporally varied from 10.6 to 527.7 m^3^, which is equivalent to 3.1–77.2 m^3^ (average 29.4 m^3^, standard deviation 21.2 m^3^) per month (Table 1). Supposing that the total projected area of all the bedrock faces of the island is 1486 m^2^, the volume change rates are equivalent to the horizontal mean erosion rates from 0.025 to 0.63 m/y (average 0.24 m/y, standard deviation 0.17 m/y), which are smaller than the previously reported cliff retreat rates in this area with a significant difference [10].

## 4. Discussion

### 4.1. Advantages and Limitations of the Proposed Methodology

Estimating the volume of changes in landforms, including landslides, gully erosion, and debris flows, has often been carried out using DEMs that are projected in two-dimensional geographical planes [34,35,36]. The use of DEMs is often advantageous because handling the data is easier and faster than 3D structured data. However, the use of geographically projected DEM is invalid for a complex object having overhanging slopes. In some cases of overhanging cliffs, vertically projected DEMs can also be used to represent the morphology and to detect the landform changes [16,37,38,39]. However, if the object has a highly complex shape like the Suzume-Jima Island, the use of vertical planes is also impractical. The comparison of 3D point clouds at different times using point-based analysis, which provides change distances between point clouds, would be a promising solution for the change detection of complex-shaped objects [25,40,41]. In such a case, smoothed point-to-mesh distances are often utilized to calculate the volume of changed areas, but still the volume estimation needs to be challenged if the missing area has a complex shape [42,43]. The methodology proposed in this study gives a straightforward workflow for the volume estimation of changed areas in complex morphology. The changed areas are represented as solid mesh polygons, whose volume is readily obtained and, furthermore, some other morphological characteristics can be investigated if the shape of each polygon is assessed in more detail, which is beyond the scope of this study.

However, as a limitation of the approach in this study, a too small volume of changed areas cannot be detected due to the errors in the point cloud registrations. Total errors affecting the quality of the data and the achievable accuracy of change detection are generally lower than the manufacturer specifications because they are derived from some different sources [44,45]. In the case of this study, the major sources of errors introduced during the data processing are internal registration errors of multiple TLS positions (2.7–31.5 mm with an RMS of 20.5 mm), georeference errors by GNSS (4.2 mm), external registration errors of the TLS point clouds in the timeline (6.4–35.9 mm with an RMS of 20.8 mm), and registration errors of UAS to TLS (24.6–39.7 mm with an RMS of 31.9 mm) (Table 1). Some other minor sources of errors during the data acquisition may be present, including range errors of laser returns by TLS (2 mm @25 m) [28] and doming distortion of point clouds by SfM photogrammetry [31], but these are small enough and negligible compared to the major error sources. The doming error by SfM photogrammetry can be negligible because the target object has a high relief and the aerial photos were mostly taken diagonally [31,46]. Based on the major sources of the errors, however, total registration errors are supposed to be on the order of 20–40 mm. We set the threshold value for the significant change detection as 80 mm based on the doubled value of the maximum registration errors, but this means that the changes smaller than this threshold cannot be detected throughout the study periods. If the changes are drastic enough by rockfalls, the proposed method would be valid, but if the changes are more gradually occurring by such as rock surface weathering, such small changes cannot be identified. During the 14 studied periods for 5.3 years, the maximum error values may accumulate up to 1.1 m (80 mm × 14), whereas the total erosion amount is equivalent to 1.3 m (0.24 m/y × 5.3 y). The measured total volume by DVE (1979.5 m^3^) may underestimate the actual erosion and weathering of the island bedrock. Although visual observations on the west land-side of the island (Figure 5c) do not support such a large amount of weathering for more than a meter, millimeter- to centimeter-scale weathering is still feasible. The actual errors of eroded and missing volume cannot be verified in the field, so the exact error estimates should be further examined in an experimental study that is beyond the scope of this study. Nevertheless, the application of the methodology to the natural island shows feasible results of minimum eroded volumes of bedrock by sea wave attacks.

### 4.2. Potential Factors Affecting the Erosion

The quinquennial analysis of the 3D point cloud data series revealed a volumetric loss of the island. According to the results, although the equivalent rate of erosion (average 0.24 m/y) of the bedrock cliffs of the island was significantly lower than the long-term erosion rate reported in the area (~1 m/y), the volumetric changes by erosion of the bedrock cliffs was rapid enough on the order of tens of cubic meters (average 29.4 m^3^) per month and the changes were mostly visible by DVE (Figure 5 and Figure 6). Since the current volume of the island on 02 October 2019 was approximately 11,300 m^3^, the island is assumed to disappear in about 30 years.

**Figure 7:**
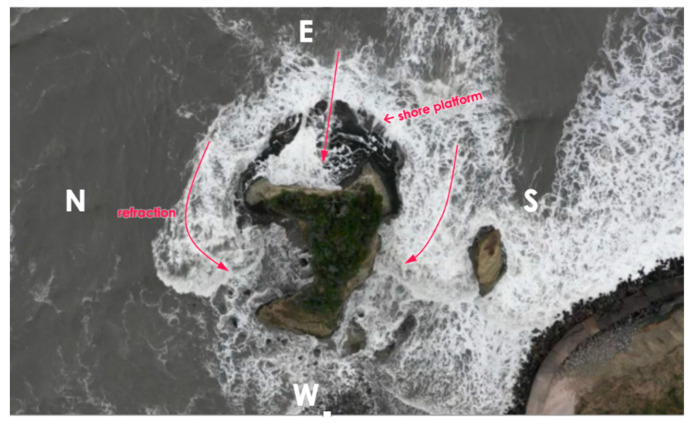
A screenshot of the video with an orthogonal view of the Suzume-Jima island from a UAV ca. 100 m above the ground. The trajectories of the tidal waves are indicated by red arrows, showing the direct attack against the eastern face of the island and indirect attacks against the northern and southern faces with the refraction of the waves.

The lower rate of the short-term (5-year) erosion than the long-term rate may be due to the complex attacks of sea waves against the small island. According to the orthogonal video view from 100 m high above the top of the island, the attacks of waves are diverted to each face of the island (Figure 7). The eastern face suffers from the most direct attacks, but the shore platform formed at the foot of the cliff seems to reduce the power of wave attacks. The northern and southern faces are affected by attacks of refracted waves which can have lower impacts than the direct attacks. The western, land-side face does not receive any significant wave attacks. Compared to coastlines having a straight shape, the diversion and complexity of wave orientations may cause weaker erosional power against the small faces of the island.

**Figure 8:**
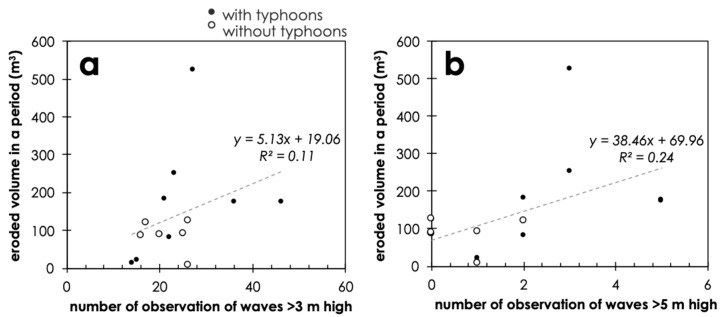
Relationships between the frequency of the high tidal waves and the eroded volume of the island. (**a**) Occurrences of offshore waves higher than 3 m. (**b**) Occurrences of offshore waves higher than 5 m. The black dots indicate the periods potentially affected by typhoons passing near the study area and the white dots indicate those without typhoon attacks.

The temporal patterns of erosion were also variable (Table 1, Figure 5). The volume of erosion averaged per month was mostly 10–40 m^3^, but in some periods (II, X, and XIII) the detected changes were low around 3–5 m^3^ per month, whereas in periods XI and XIV nearly 70–80 m^3^ of changes were observed. In the period XIV, there was a severe storm by Typhoon Faxai (hitting the area on 9 September 2020) and this likely affected the high-wave attacks and significant erosion [47]. Although every summer there have been some effects of typhoon passing nearby areas (Table 1), Typhoon Faxai was the strongest and most disastrous in the study area [48]. When compared with the occurrences of waves higher than 3 m or 5 m near the site (observation buoy Offshore Boso Peninsula), the erosion rate is slightly positively correlated (Figure 8). Although the overall correlation was not so strong and significant, it is suggested that the wave attacks have some effects on the eroded volume of the island. The effects of typhoons are not straightforward, because typhoons may be related to multiple factors including sea waves, rainfalls, and winds. The presence or absence of the typhoons during a period is therefore not so clear, but as a general trend, it can be observed that periods without typhoons tend to have a lower number of high wave observations and vice versa (Figure 8).

In Figure 8, the outlier having a large amount of eroded volume is the one affected by Typhoon Faxai. In such a case, because the time duration of the storm waves may be limited due to the rapid passing of the typhoon, the number of high wave observations can be limited, but a small number of extreme waves could have caused the severe erosion. This may also account for the lower rate of average short-term erosion. In some cases of gradually deforming cliffs, a low frequency, extreme event of cliff erosion can often exceed the amount of gradual erosion for years or decades [49,50]. Such low-frequency extreme wave attacks can also contribute to the significant deformation of the Suzume-Jima Island, and the time to the disappearance of the island may become less than 30 years, as expected by the average erosion volume. To clarify this issue, further long-term monitoring of the study site needs to be continued.

## 5. Conclusions

This study was designed for the volume-based estimate of changes in bedrock cliff along the coastline using both UAS- and TLS-derived 3D point cloud data. The application of the proposed DVE method was successful in quantifying the morphological changes of a small coastal island with registration errors of 20–40 mm, and the average erosion volumes per month was estimated to be 30 m^3^. The rate of erosion is rapid enough to keep the island for only about 30 years. Wave attacks likely have some influences on the erosion patterns both in time and space. In addition, three-dimensional structural analysis using the 3D data will also help understand the dynamic processes of the erosion of bedrock cliffs in the island.

## Figures and Tables

**Table 1:** Properties of the datasets for each field survey. Registration errors indicate the root mean square of the mean distances between the nearest points in the different point clouds. I–XIV indicate the periods between the pre- and post-change datasets that were for the calculations of the volume changes.

Num	Period	Name of Dataset	Date of Measurement	Duration (Days)	Number of Tyhoon Attacks	TLS Used	UAS Used	Number of TLS Scan Position	Internal Registration Errors for TLS (mm)	Georeference Errors (mm)	External Registration Errors (mm)		UAS to TLS Registration Errors (mm)	Eroded Volume (m^3^)	Volume per Month (m^3^)	Erosion Rates (m/y)
1	–	140624	24 June 2014	–		GLS	Phantom 2	1	–	–	13.9		33.5	–	–	–
2	I	141031	31 October 2014	129	1	–	Phantom 2	–	–	–	18.8	*	–	84.8	19.7	0.161
3	II	150211	11 February 2015	103		TX5	Phantom 2	10	5.3	–	21.2		39.7	10.6	3.1	0.025
4	III	150618	18 June 2015	127		TX5	Phantom 2	6	3.5	–	8.1		24.6	127.5	30.1	0.247
5	IV	151023	23 October 2015	127	1	GLS/TX5	Phantom 3 Pro	4	12.0	–	6.4		30.1	178.5	42.2	0.345
6	V	160223	23 February 2016	123		GLS/TX5	Phantom 3 Pro	6	15.9	4.2	–		25.1	92.3	22.5	0.184
7	VI	160618	18 June 2016	116		GLS/TX5	Phantom 3 Pro	8	14.3	–	10.6		30.2	87.9	22.7	0.186
8	VII	161029	29 October 2016	133	4	GLS/TX5	Phantom 3 Pro	5	28.1	–	25.0		33.9	185.4	41.8	0.342
9	VIII	170218	18 February 2017	112		GLS/TX5	Phantom 3 Pro	7	28.1	–	11.0		30.5	90.6	24.3	0.199
10	IX	170702	2 July 2017	134		GLS/TX5	Phantom 4	6	21.1	–	19.1		32.1	121.8	27.3	0.223
11	X	171007	7 October 2017	97	1	GLS	Phantom 4	2	31.5	–	16.7		32.0	17.0	5.3	0.043
12	XI	180127	27 January 2018	112	1	–	Phantom 4	–	–	–	35.9	*	–	254.2	68.1	0.557
13	XII	180916	16 September 2018	232	1	GLS	Mavic 2 Pro	2	30.9	–	22.9		32.1	176.6	22.8	0.187
14	XIII	190311	11 March 2019	176	1	–	Mavic 2 Pro	–	–	–	33.6	*	–	24.6	4.2	0.034
15	XIV	191002	2 October 2019	205	1	TX5	Mavic 2 Pro	4	2.7	–	23.4		35.9	527.7	77.2	0.632
								RMS	20.5		20.8		31.9			
								total						1,979.5		
								mean						141.4	29.4	0.241
								standard deviation						126.6	21.2	0.173
								maximum	31.5		35.9		39.7	527.7	77.2	0.632
								minimum	2.7		6.4		24.6	10.6	3.1	0.025
												*UAS to UAS				
